# Rapid-Acting and Human Insulins: Hexamer Dissociation Kinetics upon Dilution of the Pharmaceutical Formulation

**DOI:** 10.1007/s11095-017-2233-0

**Published:** 2017-07-31

**Authors:** Klaus Gast, Anja Schüler, Martin Wolff, Anja Thalhammer, Harald Berchtold, Norbert Nagel, Gudrun Lenherr, Gerrit Hauck, Robert Seckler

**Affiliations:** 1 Physical Biochemistry, University of Potsdam, Karl-Liebknecht-Str. 24-25, Golm, D-14476 Potsdam, Germany; 2 Sanofi-Aventis Deutschland GmbH, Industrial Park Höchst, D-65926 Frankfurt, Germany

**Keywords:** circular dichroism, dissociation kinetics, insulin analog, light scattering, rapid-acting

## Abstract

**Purpose:**

Comparison of the dissociation kinetics of rapid-acting insulins lispro, aspart, glulisine and human insulin under physiologically relevant conditions.

**Methods:**

Dissociation kinetics after dilution were monitored directly in terms of the average molecular mass using combined static and dynamic light scattering. Changes in tertiary structure were detected by near-UV circular dichroism.

**Results:**

Glulisine forms compact hexamers in formulation even in the absence of Zn^2+^. Upon severe dilution, these rapidly dissociate into monomers in less than 10 s. In contrast, in formulations of lispro and aspart, the presence of Zn^2+^ and phenolic compounds is essential for formation of compact R6 hexamers. These slowly dissociate in times ranging from seconds to one hour depending on the concentration of phenolic additives. The disadvantage of the long dissociation times of lispro and aspart can be diminished by a rapid depletion of the concentration of phenolic additives independent of the insulin dilution. This is especially important in conditions similar to those after subcutaneous injection, where only minor dilution of the insulins occurs.

**Conclusion:**

Knowledge of the diverging dissociation mechanisms of lispro and aspart compared to glulisine will be helpful for optimizing formulation conditions of rapid-acting insulins.

**Electronic supplementary material:**

The online version of this article (doi:10.1007/s11095-017-2233-0) contains supplementary material, which is available to authorized users.

## Introduction

One of the great challenges for biotechnological research is the optimum substitution of the natural regulation of the active insulin concentration in blood lost in diabetic patients by appropriate insulin analogs and advanced injection techniques. This comprises both the establishment of the basal level of insulin by long-acting insulins and the fast adjustment of the instantaneous insulin concentration in accordance to food uptake by rapid-acting insulins (RAI) (1). The present work relates to the second category of insulins. Specifically, we have compared the dissociation kinetics of the three RAI analogs lispro, aspart, and glulisine and regular human insulin (HI) from the oligomeric storage state in formulation into the monomeric state directly by measuring changes in the average mass.

The presence or fast adoption of the monomeric state is the most important precondition for the instantaneous action of insulin since only this state is biologically active. However, regular HI is monomeric only at very low concentrations (< 10^−5^ M in the presence of Zn^2+^ ions). Therefore, recombinant analogs with reduced stability of oligomers have been developed for rapid-acting purposes. On the other hand, monomeric insulins do not fulfil the conditions of long-term storage required for drug products (2). To ensure sufficient shelf life, insulin in formulation must be in a compact oligomeric (essentially hexameric) state that is resistant to chemical degradation and toxic aggregation as well. High insulin concentration and the presence of allosteric ligands, such as Zn^2+^ and phenolic compounds, favor the population of the hexameric state. Consequently, two opposing requirements, high stability of insulin in formulation and fast dissociation after subcutaneous injection play an important role for the development of RAI.

Insulin monomers consist of an A-chain (21 amino acid residues) and a B-chain (30 amino acid residues) linked by two disulfide bonds. Most information about association states of insulins in solution were originally obtained from hydrodynamic investigations at low pH (3) and neutral pH (4–7). High-resolution X-ray studies revealed the existence of different conformations of subunits within the hexameric assemblies depending on environmental conditions. Spectroscopic investigations supported the assumption that specific oligomeric structures identified in solution are consistent with the corresponding high-resolution crystal structures (8).

Nearly all insulin formulations contain phenolic substances as antimicrobial preservatives. First indications for their influence on insulin structures came from differences in the crystallization behavior. This effect was also clearly shown for insulin in solution by Wollmer *et al*. (8). Subsequent X-ray studies (9) demonstrated explicitly the changes from hexamers in the 2 Zn^2+^-structure to the structure specific in the presence of phenol. At the same time, known canonic hexamer structures of insulin in the absence and presence of allosteric ligands, such as bivalent metal ions (Zn^2+^, Co^2+^), lyotropic anions (Cl^−^, SCN^−^), and phenolic ligands (phenol, m-cresol) were classified as T_6_, T_3_R_3_, and R_6_ (10) (9). The R_6_-hexamer in the presence of phenol was reported to be the thermodynamically most stable species. More structural details of the R_6_ human insulin hexamer in solution were provided by NMR spectroscopy and restrained molecular dynamics (11). The solution structure resembles the X-ray structure. However, the extension of the α-helix in the B-chain, a characteristic of the R_6_ state, is shorter in the solution structure.

Lispro was the first formulated RAI (Humalog®) on the market. The primary structure of lispro differs from that of HI by an inversion of the amino acids Pro^28^ and Lys^29^ in the sequence of the B-chain. The crystal structure obtained under formulation conditions displays a T_3_R_3_^f^ conformation (12). However, there is evidence that the structure in formulation is rather the R_6_ conformation (13). This should be expected since the formulation contains both Zn^2+^ ions and m-cresol at sufficiently high concentrations.

The R_6_ conformation was indeed observed in crystals of the RAI aspart grown in the presence of either phenol or m-cresol (14). In the case of aspart, Pro^28^ of the B-chain is substituted by Asp. Formulations of Aspart (NovoRapid®) contain equal amounts of phenol and m-cresol (Supplemental Table I). Recently, the pharmacokinetics and pharmacodynamics of insulin aspart could be improved using formulations (called faster-acting insulin aspart) containing the additional excipients niacinamide and L-arginine (15,16).

The amino acid sequence of the third RAI glulisine differs from the HI sequence at positions 3 and 29 of the B-chain. Asn^3^ is substituted by Lys and Lys^29^ by Glu. In contrast to Humalog® and NovoRapid®, formulations of glulisine (Apidra®) contain no Zn^2+^ (Supplemental Table I). In the absence of Zn^2+^ ions, specific differences in the solution structure and the dissociation behavior compared to Zn^2+^-stabilized insulin structures can be expected. To the best of our knowledge, the high-resolution structure under conditions corresponding to Apidra® formulation has not been published so far. Known structural and functional properties of glulisine have been reviewed by Becker (17).

Direct investigations of the kinetic changes of the association state in terms of the mass of oligomers have not been published so far. Techniques yielding the desired information (light, x-ray, and neutron scattering, hydrodynamic investigations including size exclusion chromatography) are slow in general and require relatively high protein concentrations. Up to now, information about the dynamics of insulin assemblies and the kinetic transitions between different oligomeric states was obtained only using indirect (mostly optical spectroscopic) signals (10,18–21). Particularly, very different rates of dissociation of T_6_ hexamers (10) and R_6_ hexamers (19) have been observed.

The purpose of our studies is a comparison of the dissociation behavior of the three RAI and HI. Specifically, we wanted to elucidate biophysical properties of the insulins potentially important for their dissociation and absorption after injection, but we do not aim at modeling or simulating the corresponding processes under *in vivo* conditions.

We initiated oligomer dissociation by changing both, insulin concentration and solvent composition (medium) and we monitored structural changes using static light scattering (SLS), dynamic light scattering (DLS), and circular dichroism (CD). The most direct measure of the association state is the average molecular mass, which we obtained from static light scattering. To further characterize initial, transient and final states, we used DLS to determine hydrodynamic Stokes radii (R_S_) and we measured CD signals. Combination of hydrodynamic dimensions, R_S_, with the corresponding masses gives an idea of the compactness of insulin oligomers or aggregates in different conformational states. The near-UV CD data yield information about extent and time dependence of changes in tertiary structure. This is of particular importance, as the near-UV CD spectra can be used to distinguish between T_6_, T_3_R_3_ and R_6_ conformations of insulins (22).

Two main experimental schemes were used. Scheme I is characterized by a substantial dilution of the insulins with an appropriate solvent facilitating pronounced changes in the association state. To reach this, we have rapidly diluted RAI formulations with PBS. This simple experimental scheme provides information about basic differences concerning the dissociation kinetics and the final association states and on the influence of formulation excipients. This type of investigations yields the “speed limit” of the dissociation process and may be considered to reflect the dilution conditions after intravenous injection.

Scheme II is derived from the conditions after subcutaneous injection. In this case, we study the dissociation processes at insulin concentrations corresponding to an only two-fold dilution of U100 formulations and at various m-cresol concentrations, decreased stepwise from the formulation concentration. This scheme may be considered to simulate the conditions in the bolus formed after injection, where a continuous disappearance of phenolic additives is expected to occur after an initial moderate dilution of the insulin formulation. The rapid diffusion of phenolic additives after injection is considered as an essential factor influencing insulin dissociation kinetics and the entire absorption process in the literature (2,12,23).

## Material and Methods

### Materials and Sample Preparation

For studies on insulins under formulation conditions we have used the marketed U100 formulations Humalog® (Lilly, Indianapolis, USA), NovoRapid® (Novo Nordisk, Bagsveard, Denmark), as well as Apidra® and Insuman Rapid® (Sanofi-Aventis, Frankfurt, Germany). The compositions of the formulations are shown in Supplemental Table I. Lyophilized samples of the insulins (lispro, aspart, glulisine, and HI) and solvents to the corresponding formulations (placebos) were provided by Sanofi-Aventis (Frankfurt, Germany). PBS (10 mM buffer) was prepared from tablets (Calbiochem, Germany). Glycerol (85%), phenol and m-cresol were provided from Roth (Germany). ZnCl_2_ was supplied from Merck (Germany). All chemicals were of analytical grade. Solvents were prepared using Milli-Q water (Merck Millipore, Germany) and were filtered and degassed after preparation using 0.45 μm Millicup filter units (Merck Millipore, Germany). In special cases, dialysis against the corresponding solvent using dialysis tubing (MW cut-off 3500, Spectra/Por©, Spectrum Laboratories, USA) was done ensuring the specified solvent conditions. Dialysis was always done, if stock solutions with high insulin concentrations were used. For measurements in Zn^2+^-free PBS, EDTA was added to the stock solutions after dissolving the drug substance, in order to remove Zn^2+^ completely. Afterwards, the samples were dialysed against PBS.

Samples for light scattering were filtered either through 0.1 μm Whatman-Anotop filters (VWR, Germany) directly into 3-mm-pathlength micro-fluorescence cells (105.251-QS, Hellma, Germany) immediately prior to use or were subjected to ultracentrifugation. Usually, about 500 μl were centrifuged at 75000 g for 30 min. An amount of 80–150 μl of the supernatant was quickly transferred into the sample cell. Peptide concentrations were determined photometrically using a specific absorption A(276 nm, 1 cm pathlength, 1 mg/ml) of 1.08.

### Choice of Dilution Media and Dilution Jumps

An ideal dilution medium has to combine two requirements: to be suitable for the chosen biophysical methods and to be consistent with physiological environments. The first step during application of insulins is the injection into the subcutaneous tissue (ST). Therefore, the initial solvent change is a mixture of formulation with the interstitial fluid (IF). IF (see Supplemental Table II) is not a suitable medium because of the high protein content. Thus, one has to select a substitute, which is similar to the IF at least concerning essential solvent properties as ionic strength, kinds of ions and pH. In addition to Na^+^ and K^+^ the IF contains the bivalent metal ions Mg^2+^ and Ca^2+^ that could affect the association state of insulins, as it was shown for Ca^2+^ (24,25). Therefore, we tested possible influences of MgCl_2_ and CaCl_2_. The presence of 0.6 mM MgCl_2_ had practically no effect on the association state of the investigated insulin analogs. However, dilution of formulations with PBS containing additionally CaCl_2_ (final concentration 1.6 mM after mixing) led to a rapid formation of huge amounts of large aggregates in the case of lispro and aspart (data not shown). This impedes the use of simulated IF composition, since it cannot be applied to all analogs in the present study. Therefore, we decided to use PBS as an adequate dilution medium for a comparison of the dissociation behavior of the RAI analogs.

The technique of mixing formulation with dilution medium strongly determines the feasibility of dissociation experiments as well as the relation of the results to physiological processes. From a biophysical point of view, the applied changes in the solution conditions must lead to clearly measurable changes in the association state towards the biologically active monomeric state. This was realized by scheme I, which uses sufficiently large protein concentration jumps caused by dilution with solvent (PBS). By adding particular amounts of excipients to the solvent, concentration changes of excipients upon dilution can be compensated or modified in order to measure the influence of excipients on dissociation. The dilution factor is limited by the sensitivity of the biophysical methods. Taking into account the concentration of U100 insulin formulations of 3.5 g/l and a low-concentration limit for light scattering investigations of insulins of 0.1–0.2 g/l, a dilution factor of 20 is feasible. We have mostly used 20-fold dilution to determine the maximum speed of dissociation during one-step dilution experiments. Furthermore, this scheme is useful to measure the influence of excipients on the dissociation kinetics and the final association state after dilution. In order to vary the final concentrations of excipients over a wide range we have applied other dilution factors and modified formulations with higher insulin concentrations in some cases.

*In vivo*, the immediate dilution factor upon subcutaneous injection is assumed to be only about two (23). Such a weak protein dilution does not considerably change the association state. However, the decrease in the concentration of excipients due to fast diffusion can alter the association state rather quickly in an alternative way. This is of particular relevance for phenolic additives. Their influence will be considered in more detail in the case of m-cresol. In order to take into account this pathway, we have used experimental scheme II (Fig. 11) to imitate twofold dilutions of U100 formulations together with a subsequent decrease in the concentration of m-cresol. This procedure is based on the presumption that the final equilibrium state and the kinetics after dilution depend only on final solution conditions and on identical initial states prior to dilution. The reference condition for the experiments is insulin U100 twofold diluted with PBS. Following scheme II, the insulin concentration is kept constant while the m-cresol concentration is lowered stepwise. Scheme II uses modified formulations (U900) with nine-fold higher insulin concentration (32 g/l) and an m-cresol concentration of 29 mM, which are 18-fold diluted with PBS containing defined amounts of m-cresol. The identity of the initial states in U900 and U100 conditions was tested in equilibrium light scattering and CD experiments. The m-cresol concentration can thus be lowered stepwise down to 1.6 mM (Fig. 11). In order to further decrease the m-cresol concentration, we have also used dialysis against media with the corresponding low m-cresol content.

### Circular Dichroism (CD)

CD measurements in the near-UV region were done in a Jasco J-715 spectropolarimeter equipped with a Peltier-thermostat controlled cell holder using quartz cuvettes with appropriate pathlengths between 0.1 mm and 10 mm (Hellma, Germany). The instrument was calibrated using 1S-(+)-10-camphorsulphonic acid (26). After baseline correction, measured ellipticities θ were converted into mean-residue ellipticities [θ] using the mean-residue weights of 113.6 g/mol for HI and lispro, 114.0 g/mol for aspart, and 114.2 g/mol for glulisine, respectively. The CD measurements in formulations and diluted formulations were complicated considerably by strong absorption of the phenolic excipients. Because both, the concentrations of phenolic additives and the insulins were varied, it is difficult to specify general detection limits. Kinetic experiments were triggered by manual mixing within the sample cells.

### Equilibrium and Kinetic Measurements of Static and Dynamic Light Scattering

Simultaneous static and dynamic light scattering measurements were done with the same instrument at a scattering angle of 90°. The custom-built apparatus, equipped with a diode-pumped continuous wave laser (Millennia IIs, Spectra-Physics) and a high quantum yield avalanche photodiode, has been described in detail (27). Instead of using a commercially available stopped-flow mixing device (27), manual mixing (dead time about 10 s) was preferred in the present work. Manual mixing ensures stable solution composition, particularly during long-term experiments.

A primary data accumulation interval T_acc_ = 8 s was used for all SLS and DLS experiments. T_acc_ defines the time resolution that is consistent with the dead time and yields reasonable (short) time averages of the mean scattering intensity I(T_acc_) and the time-autocorrelation function ACF(T_acc_) of the fluctuations in the instantaneous scattering intensity. Hundreds of pairs of I(T_acc_) and ACF(T_acc_) were stored transiently before calculating kinetic and equilibrium data. Fig. 2 shows an example of an original data trace of scattering intensity. Translational diffusion coefficients D were obtained from the measured autocorrelation functions using the program CONTIN (28). CONTIN yields intensity distribution functions I(D), which can be calculated without further assumptions concerning the morphology of the particles. Diffusion coefficients were converted into Stokes radii via the Stokes-Einstein equation R_S_ = k_B_T/(6πηD), where k_B_ is Boltzmann’s constant, T is the temperature in Kelvin, and η is the solvent viscosity. In our work, the hydrodynamic quantities are presented in terms of R_S_. Viscosities were measured using an Ubbelohde-type viscometer (Viscoboy-2, Lauda, Germany).

Apparent molecular masses, M_app_, were calculated from the relative excess scattering intensity, *I*_*exc,rel*_ defined as *I*_*exc* , *rel*_ = (*I*_*solution*_ − *I*_*solvent*_)/*I*_*reference*_, where I_solution_, I_solvent_ and I_reference_ are the scattering intensities of solution, solvent, and reference scatterer (toluene, in our case), respectively. M_app_ is related to *I*_*exc,rel*_ by *M*_*app*_ = *k*_*opt*_⋅*I*_*exc* , *rel*_/*c*, where c is the peptide concentration and k_opt_ is an optical constant depending on physical quantities of the scattering experiment as scattering angle, wavelength, reference sample, refractive index n of the solution and refractive index increment (dn/dc) of the proteins. A (dn/dc) of 0.19 ml/g was used for all insulins in the individual solvents. The size distribution obtained with CONTIN was additionally used to separate the scattering intensity of insulin in the association equilibrium from that of other scattering particles.

The notation M_app_ must be used for the following reasons. Extrapolation to zero insulin concentration to eliminate the influences of intermolecular interactions (virial effects) on the true molecular mass is not adequate because we wanted to study the association equilibrium of the insulins. However, significant virial effects in addition to changes caused by shifts in the association state became evident only at concentrations above 10 g/l under the investigated solvent conditions. Possible influences on the measured association state will be discussed in particular cases.

Furthermore, only the term M_app_ is correct, as the measured mass is mostly an average over different association states. It is even more appropriate to present relative apparent molecular masses M_rel_ = M_app_/M_mon_. M_mon_ is the monomer mass calculated from the amino acid sequence.

### Use of Power Laws for Estimations of Compactness

Data on the association states of the different insulins are derived from the apparent molecular masses measured by SLS. Stokes radii measured by DLS depend on both the state of association and the shape of oligomers, particularly the packing of the monomeric subunits within the oligomer. The Stokes radius is not unequivocally related to the association state.

General information about the compactness of oligomers can be obtained from the relation between M_app_ and R_S_. This can be done using known scaling laws for the link between hydrodynamic properties (e.g. R_S_) and the corresponding mass for particular structural classes (29,30). The general form of such a scaling law is R_S_ = a^.^M^b^. The coefficients for any structural type can be obtained from a sufficiently large number of related pairs M, R_S_. For proteins such scaling coefficients have been obtained for the globular (compactly folded) and the fully unfolded state (proteins lacking disulfide bonds at high denaturant concentrations) (30). A plot of R_S_ versus M in double-logarithmic scale for both cases is shown in Fig. 7 (solid and dashed lines). This representation is particularly useful to classify the overall structural type of a protein, peptide or peptide aggregate with unknown high-resolution structure in solution. The positions of the corresponding data pairs M, R_S_ in the diagram provide information about the molecular dimensions of the molecule or the molecular assembly.

## Results

### Equilibrium States of Rapid Acting Insulins and Human Insulin

**Fig. 1:**
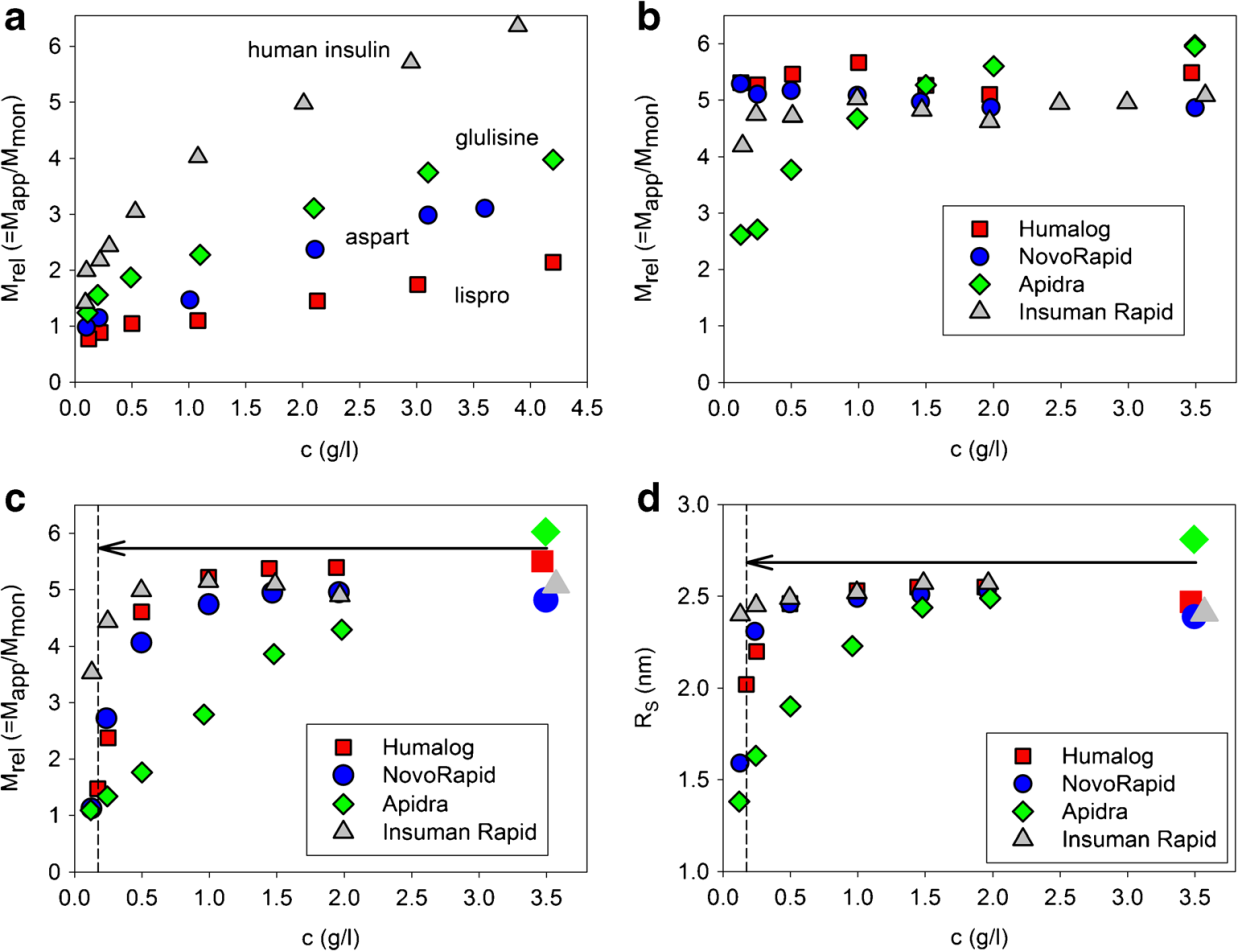
Association equilibria (M_rel_ = M_app_/M_mon_) of lispro, aspart, glulisine and human insulin (**a**) in 10 mM PBS, pH 7.4 in the absence of Zn^2+^ and phenolic ligands and (**b**) under formulation conditions with constant concentrations of phenolic ligands as specified in Supplemental Table I. (**c**) Average relative molecular masses M_rel_ = M_app_/M_mon_ and (**d**) Stokes radii R_S_ of the four insulins in formulation at c = 3.5 mg/ml before dilution (large unframed symbols) and after dilution of formulation with PBS, pH 7.4 (edged symbols), T = 23°C. The ratio of insulin to Zn^2+^ and phenolic ligands is kept constant throughout the dilution series. The arrows depict the 1:20 dilution jump applied during scheme I kinetic experiments. Error bars indicating typical deviations (±10% for M_rel_ and ±3% for R_S_) are omitted in this and subsequent figures for clarity.

Before measuring the dissociation kinetics of the insulins, equilibrium association states under various conditions, initial states in formulations and final states after dilution were characterized by equilibrium measurements. The association equilibria under near physiological (PBS with no added Zn^2+^) and formulation solvent conditions are shown in Fig. 1a and b, respectively. Although the results in Fig. 1a indicate a strong tendency of all insulins to dissociate with decreasing concentration in saline solution, the differences caused by modifications in the amino acid sequence are evident. The higher stability of oligomeric structures in formulation solvents becomes obvious by comparing the association states in Fig. 1a and b.

The association states after mixing different amounts of formulation and PBS are shown in Fig. 1c. The concentration dependence differs considerably from that in PBS and cannot be considered as a simple association equilibrium, as both insulin concentration and solvent composition are changed by mixing formulation and buffer. The association states at insulin concentrations of 0.17 mg/ml marked by a dashed vertical line correspond to the final states that are approached during kinetic experiments using 1:20 dilution jumps. The values of M_rel_ close to one indicate that all RAI approach the monomeric state under those dilution conditions. The association state of HI after 1:20 dilution is still far away from the monomeric one. The large difference between the states of HI in PBS (Fig. 1a) and after dilution of the formulation with PBS (Fig. 1c) demonstrates the strong Zn^2+^ binding and consequently the higher stabilization of oligomers in the case of HI.

Fig. 1d shows the corresponding changes in R_S_ after dilution of formulations with PBS. The dissociation of oligomers is also indicated by the decrease in R_S_. However, Stokes radii cannot be directly related to the number of monomers forming the oligomers. Nevertheless, Stokes radii are useful to estimate the compactness of oligomeric states in particular cases.

### Kinetics of Changes of Relative Molecular Masses

**Fig. 2:**
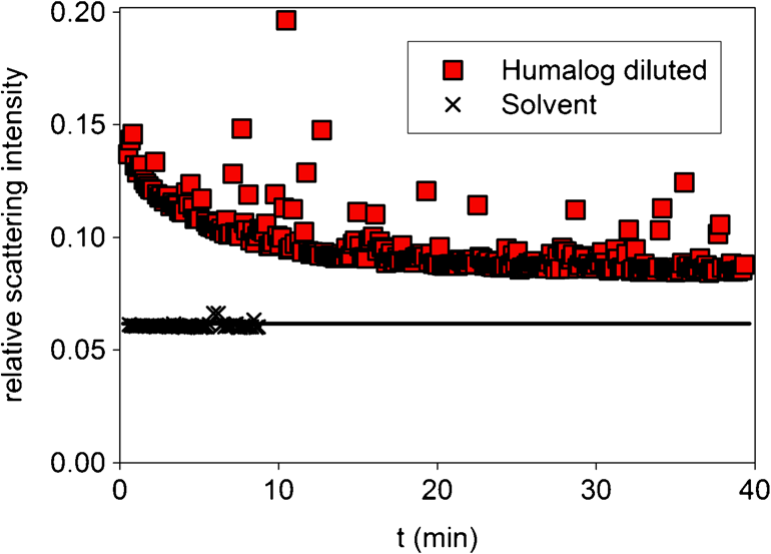
Changes of the relative light scattering intensity after a 1:20 dilution jump of Humalog® with PBS, pH 7.4, T = 23°C (final insulin concentration 0.18 mg/ml). For comparison, we have also shown the scattering intensity of the corresponding solvent (1:20 dilution of placebo with PBS).

Initially, all systematic kinetic light-scattering experiments were done using 1:20 dilution jumps of U100 formulations with PBS. Figure 2 shows the original data of the temporal changes in the light-scattering intensity observed for Humalog®. The changes in scattering intensity level off after about 40 min. The intensity pattern is typical of the majority of experiments. Note the low excess scattering of the solution over that of the solvent. The low excess scattering and the unavoidable contribution of spurious scattering were the main problems involved in these investigations. A special data treatment procedure had to be applied to derive relative masses, M_rel_ = M_app_/M_mon_, from the primary data.

**Fig. 3:**
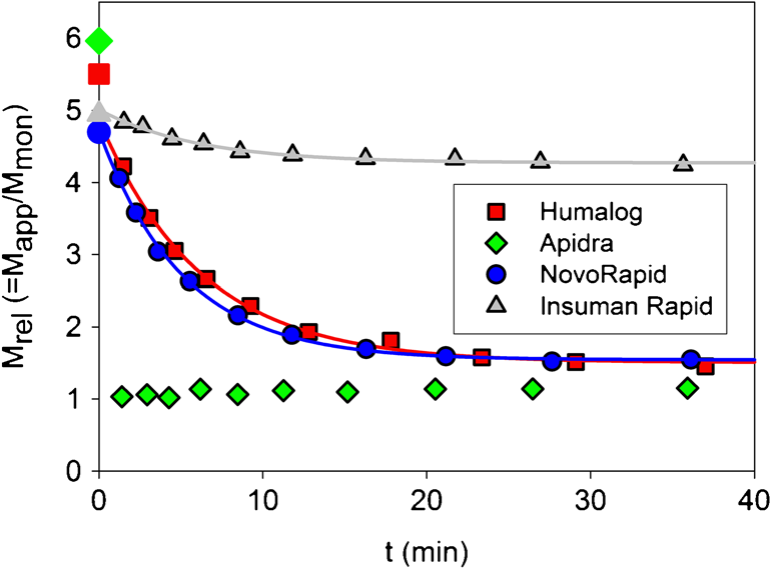
Changes of the average relative molecular masses M_rel_ after 1:20 dilution jumps of formulations with PBS, pH 7.4, T = 23°C. The large open symbols at *t* = 0 indicate the initial M_rel_ in formulation measured in equilibrium experiments. The continuous lines show single-exponential fits to the experimental data (for results see Table I).

The changes in M_rel_ of the RAI and HI after 1:20 dilution jumps are shown in Fig. 3. Between 10 and 20 original data acquisition intervals had to be accumulated in order to improve the (S/N) ratio of the intensity time-autocorrelation functions. The loss in the number of data points had to be accepted in favor of obtaining size distributions allowing the correct estimation of the scattering from insulin oligomers. Interestingly, dissociation of insulin glulisine into monomers is complete within the dead time of the experiment. In contrast, insulins lispro and aspart dissociate much more slowly. Both insulins approach a quasi-monomeric state with comparable rates. Some dissociation of human insulin also occurs on the same time scale of a few minutes. However, the final dissociation steady state reached by HI is far away from the monomeric one, as expected from the equilibrium studies. All kinetic traces could be fitted by a sum of one exponential and a constant background within the experimental error (solid lines in Fig. 3).

### Near-UV CD Spectra in Equilibrium States and Kinetic Changes of the Ellipticities at Characteristic Wavelengths

**Fig. 4:**
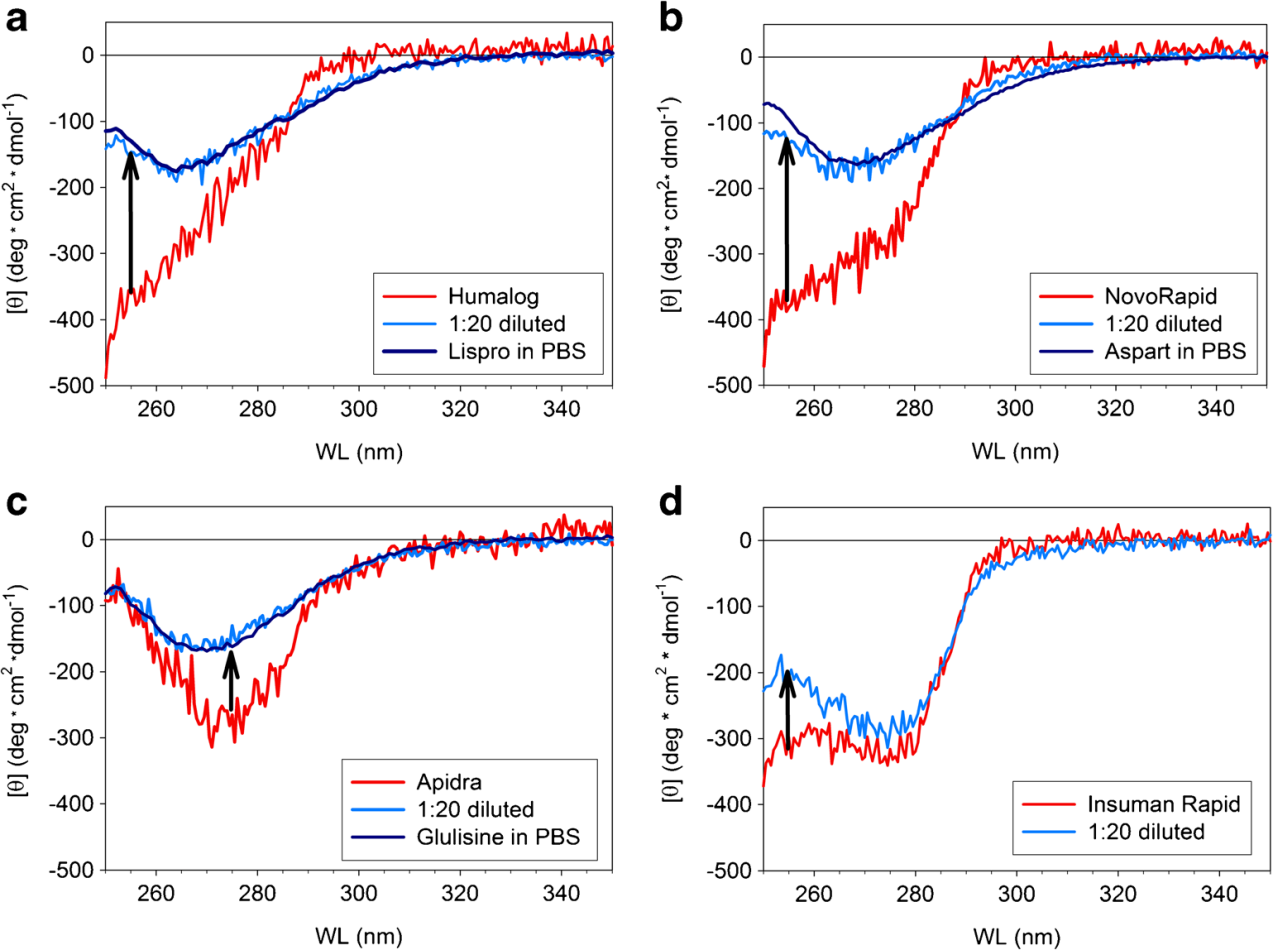
Near-UV CD spectra obtained for (a) Humalog®, (b) NovoRapid®, (c) Apidra®**,** and (d) Insuman Rapid®. All figures additionally contain the respective spectra after 1:20 dilution of the formulations with PBS, pH 7.4, T = 23°C (*light blue*) and of the insulin dissolved in PBS, pH 7.4, T = 23°C (*dark blue*, RAI only). The arrows indicate the expected signal changes at 255 nm (**a, b, d**) or 275 nm (**c**) during kinetic experiments.

The equilibrium near-UV CD spectra of the RAI and HI in formulation, after 20-fold dilution with PBS and in PBS are shown in Fig. 4. Knowledge of the changes in near-UV CD accompanying the dissociation process is indispensable in order to distinguish different types of hexamers and to estimate the time scales of changes in tertiary structure. For example, strong negative ellipticities in the wavelength range between 250 and 265 nm are typical of insulin in the R_6_ state observed in the presence of both Zn^2+^ and phenolic additives (8,13). Furthermore, consideration of equilibrium CD spectra is important for the choice of wavelengths for monitoring the kinetic CD changes. According to the initial and final spectra, the kinetic traces were recorded at 255 nm for the formulations Humalog®, NovoRapid® and Insuman Rapid® and at 275 nm in the case of Apidra®.

**Fig. 5:**
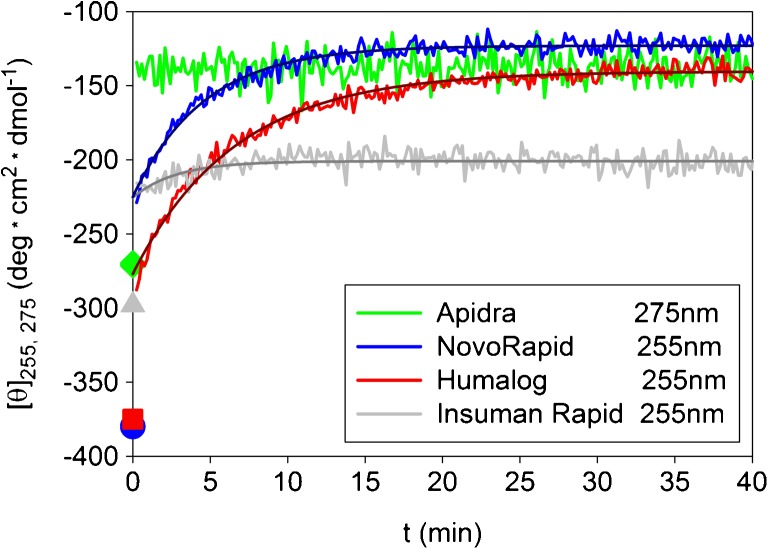
Changes of the specific ellipticities after 1:20 dilution jumps of formulations with PBS, pH 7.4, T = 23°C. The symbols at *t* = 0 indicate the specific ellipticities in formulation measured during equilibrium experiments. The observable kinetic traces for Humalog®, NovoRapid® and Insuman Rapid® could be fitted by single-exponentials within the experimental error (for results see Table I).

The time course of the CD signal at the selected wavelengths after 1:20 dilution jumps is shown in Fig. 5. Some features resemble the dissociation kinetics observed in light-scattering experiments. Again, very fast changes completed within the experimental dead time were observed for Apidra®. The changes in the cases of Humalog® and NovoRapid® clearly include a slow component. Insuman Rapid® shows also slow, but only weak changes within the accessible time range. Interestingly, remarkable dead time amplitudes have been measured in the case of Humalog®, NovoRapid® and Insuman Rapid®, which do not have its counterpart in the changes of relative masses. As in the case of light scattering the kinetic records could be fitted by one exponential and a constant.

### Summary of Standard Dilution Data, Dependence of the Dissociation Rates on the Final Concentration of Excipients

Characteristic parameters of the kinetic transitions for the 1:20 dilution are summarized in Table I. Aspart and lispro approach a final state that is largely monomeric indicated by M_rel_ ~ 1.5, while glulisine is completely dissociated (M_rel_ = 1). There are only small differences between the time constants obtained for lispro and aspart. Additionally, the reaction times obtained by light scattering and CD agree within the experimental error. In the case of human insulin, the reaction time constants are of the same order of magnitude as for lispro and aspart. The difference between the time constants obtained for HI by CD and light scattering is probably due to the large experimental errors caused by the small kinetic amplitudes and the low (S/N) ratio of the kinetic records.

**Table I:** Summary of Kinetic Data after 1:20 Dilution of Formulations with PBS According To Scheme I

Formulation	Mean oligomeric state in formulation (M_app_/M_mon_)	Mean oligomeric state after dilution (M_app_/M_mon_)	τ_LS_ (min)	τ_CD_ (min)
Apidra®	6.0	1.0	< 0.3	< 0.2
Humalog®	5.5	1.5	6.2 ± 1.0	6.4 ± 0.5
NovoRapid®	4.7	1.5	5.1 ± 1.0	4.6 ± 0.5
Insuman Rapid®	4.9	4.2	6.0 ± 2.0	2.8 ± 2.0

**Fig. 6:**
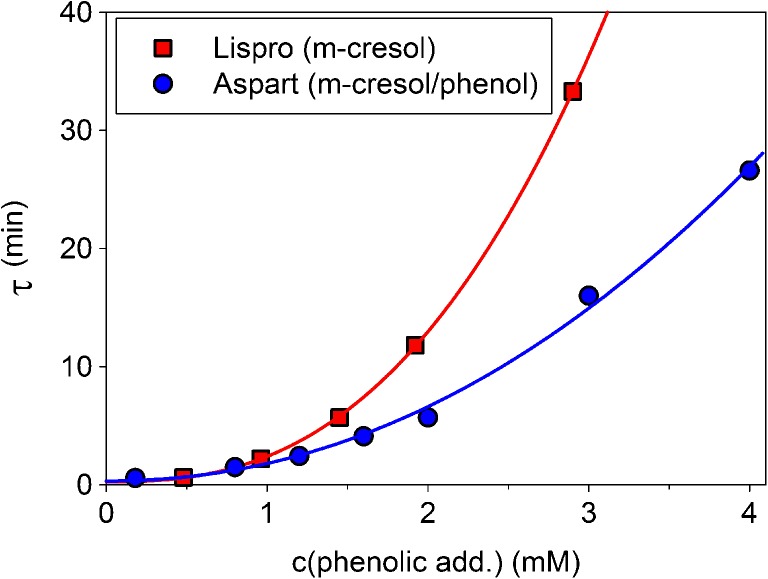
Dependence of the observable dissociation time τ on final concentration of phenolic additives after dilution for lispro and aspart. Humalog® contains 29 mM m-cresol, while NovoRapid® contains 16 mM m-cresol and 16 mM phenol. The methods for variation of final concentration are described in Materials and Methods. For the calculations of τ, we have used the light scattering results and fitted the concentration dependence by an empirical power law τ(c) = τ_0_ + a_*_c^b^ .

Since phenolic additives stabilize the hexameric states of insulins considerably, systematic investigations of the influence of phenolic excipients on the dissociation kinetics were done up to final excipient concentrations of 4 mM. The results are shown in Fig. 6. The influence is remarkable, for both insulin lispro and aspart. The same increase of the concentration of phenolic additives had no measurable effect on the fast dissociation of glulisine hexamers (data not shown).

The influence of further additives appeared to be negligible. Results of investigations on the influence of excess zinc are summarized in Table II. No influence was found concerning the presence of glycerol (data not shown).

**Table II:** Influence of Zn^2+^ on Dissociation Times

sample	final Zn^2+^ concentration after dilution (mM)	final Zn^2+^/insulin monomer molar ratio	τ_CD_ (min)
Humalog® (1**:20**)	0.015	0.5	6.4 ± 0.5
	0.2	6.7	7.2 ± 0.5
NovoRapid® (1**:20**)	0.015	0.5	4.6 ± 0.5
	0.2	6.7	6.9 ± 0.5

Dissociation times determined from CD data, the higher Zn^2+^ concentrations were produced by adding Zn^2+^ to the dilution buffer

### Consequences of the Absence and Presence of Zn^2+^ and Phenolic Additives on Insulin Association States and Dissociation Kinetics

The investigations aim to understand the different self-association/dissociation properties of these insulins combined with the influence of relevant excipients. The results show the influence of specific modifications of the formulation conditions of lispro, aspart, glulisine and HI on the association states and the dissociation kinetics.

**Fig. 7:**
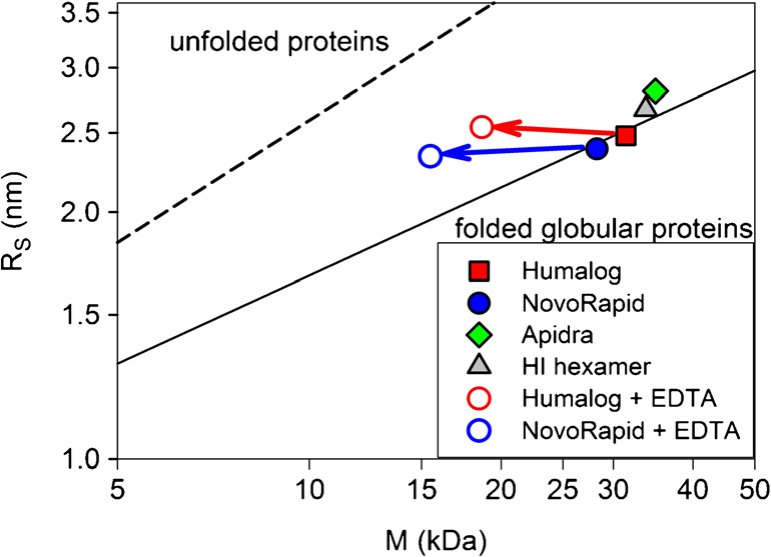
Compactness of insulin hexamers in Humalog®, NovoRapid®, Apidra® and as reference HI, c = 4.0 mg/ml, in PBS, 0.45 mM ZnCl_2_, pH 7.4, T = 23°C (filled symbols). Empty symbols display structural consequences of the removal of Zn^2+^ from the formulations Humalog® and NovoRapid® by adding 0.6 mM EDTA. The simultaneous changes in average molecular mass and average Stokes radius R_S_ can be conveniently displayed in a diagram R_S_ versus M in logarithmic scales. Addition of EDTA to Humalog® and NovoRapid® results in a shift to smaller masses while R_S_ are essentially unchanged. The average compactness according to the scaling laws for compactly folded globular proteins and proteins (without disulphide bonds) unfolded by guanidinium chloride are shown for comparison.

Figure 7 shows the effect of removing Zn^2+^ from the formulations of Humalog® and NovoRapid® on the average molecular mass and the Stokes radius. This was done by adding EDTA, thus bringing the formulation conditions of Humalog® and NovoRapid® closer to that of Apidra®. In both cases, the stable and compact hexameric state dissociates slowly (kinetic data not shown) into smaller assemblies according to the average mass. Remarkably, this is not accompanied by a measurable decrease in the hydrodynamic dimension for both insulins. Accordingly, the presence of Zn^2+^ is crucial for the formulations Humalog® and NovoRapid® in order to obtain compact oligomeric states. These general observations render further modifications of Humalog® and NovoRapid® inadequate.

**Fig. 8:**
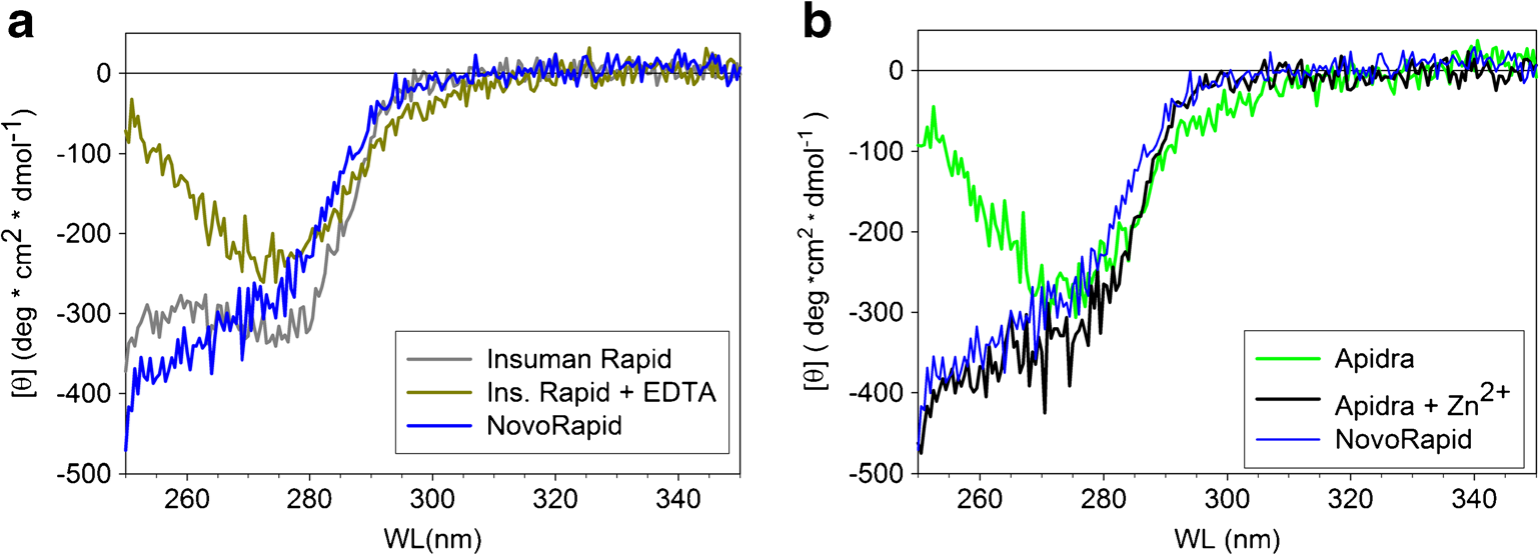
Influence of Zn^2+^ on the tertiary and quaternary structures of insulin glulisine and HI. (**a**) Removal of Zn^2+^ from formulation Insuman Rapid® by adding 0.6 mM EDTA diminishes the strong negative CD amplitude characteristic of the R_6_ structure. (**b**) Conversely, addition of 0.25 mM ZnCl_2_ to formulation Apidra® converts the shape of the CD spectrum to that observed in the case of the R_6_ configuration. For comparison, we have also shown the CD spectrum of NovoRapid® having a shape typical of the R_6_ state.

**Fig. 9:**
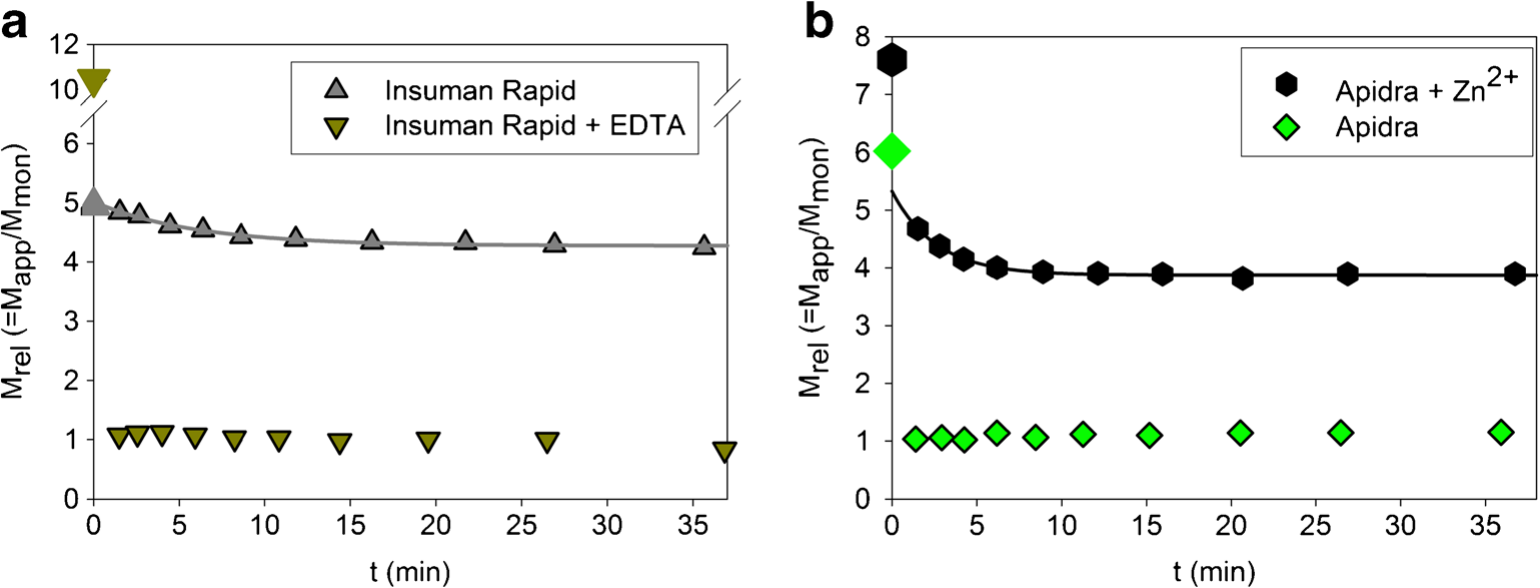
Influence of Zn^2+^ on the dissociation kinetics of insulin glulisine and HI. (**a**) Removal of Zn^2+^ from Insuman Rapid® by adding 0.6 mM EDTA makes the dissociation as fast as in the case of Apidra®. However, the large initial mass hints at an association behavior that differs from that of Apidra® (see also Fig. 10). (**b**) On addition of 0.25 mM ZnCl_2_, Apidra® totally loses its fast dissociation properties directly into monomers. Moreover, an initial association state comprising assemblies larger than hexamers is observed. The first step of the kinetics is a fast dissociation towards hexamers, which is followed by slow dissociation into oligomers with an average mass corresponding to four monomers.

Figs. 8 and 9 demonstrate differences and similarities between insulin glulisine and HI by adding or removing Zn^2+^. According to Fig. 8a, removal of Zn^2+^ transforms HI from a structure in a phenol stabilized hexamer (see also the spectrum of aspart in formulation) to a state that is characterized by a CD spectrum similar to insulin glulisine in Apidra® formulation. On the other hand, addition of Zn^2+^ to the Apidra® formulation converts glulisine into a structure with a near-UV CD spectrum nearly identical to that of aspart in NovoRapid® formulation (Fig. 8b).

Fig. 9a and b show the consequences of the same changes on the association state and the dissociation kinetics. After complete removal of Zn^2+^ from the Insuman Rapid® formulation, HI dissociates into monomers as fast as glulisine in Apidra® (Fig. 9a). However, agglomeration of HI into structures larger than hexamers is observed under the modified starting conditions. On the other hand, addition of Zn^2+^ to Apidra® supports the formation of larger assemblies in formulation and brings the dissociation behavior close to that of HI in Insuman Rapid® (Fig. 9b).

**Fig. 10:**
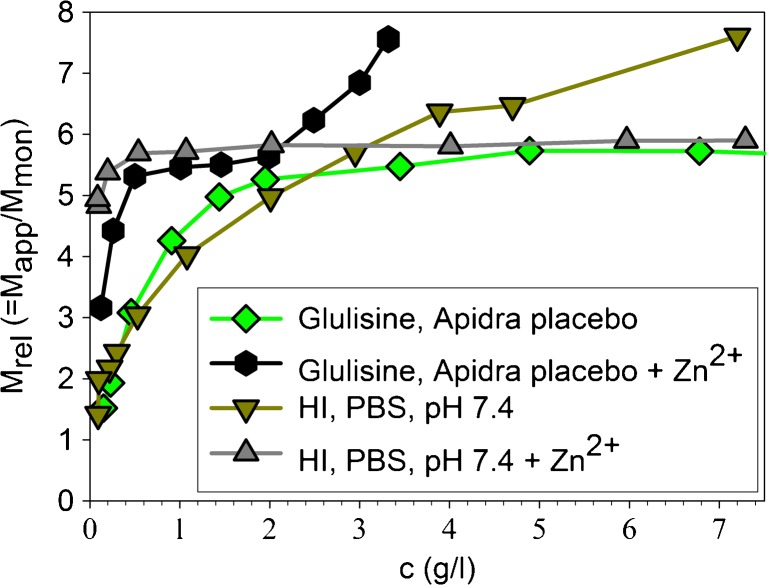
Influence of Zn^2+^ on the association behavior of glulisine in placebo to Apidra®. The association equilibrium is strongly shifted towards hexamers at low concentrations. Second, formation of assemblies larger than hexamers becomes evident at concentrations above 2 mg/ml. This behavior is totally different from that of HI in the presence of Zn^2+^. For comparison, we have also shown the association equilibria of HI in PBS with and without Zn^2+^.

To get further structural information on the state of glulisine in Apidra®, we have done additional experiments. Figure 10 gives evidence for significant changes in the association equilibrium of glulisine, if Zn^2+^ is added to the formulation, and shows the effect of the inverse procedure in the case of HI, namely the removal of Zn^2+^ from the formulation Insuman Rapid®.

In the presence of Zn^2+^ and at concentrations below 2 mg/ml, oligomers of both insulins exhibit similar stabilities according to the association equilibria. However, insulin glulisine shows a tendency to form larger assemblies above 2 mg/ml. HI does not form such structures under the same conditions.

In the absence of Zn^2+^ and low concentrations, insulin glulisine and HI populate association states of nearly the same size. Remarkably, the size of glulisine assemblies levels off close to that of hexamers at high concentrations, whereas the size continuously increases in the case of HI. Differences between the large oligomeric structures of glulisine and HI will be considered in more detail in the discussion.

The compactness of the investigated insulins in their formulations is compared in Fig. 7 by plotting the Stokes radii versus the average mass in double logarithmic scale. For better illustration, we have shown the lines corresponding to the scaling equations between mass and Stokes radius for unfolded and folded proteins. All insulins are essentially as compact as typical globular proteins under their particular formulation conditions. Notably, this holds also for glulisine, despite a possibly different structural organization of hexamers compared to the R_6_ states of HI, insulin lispro and insulin aspart (see *Discussion*).

### Impact of m-Cresol on the Dissociation Behavior of Lispro under Subcutaneous Injection-like Conditions

**Fig. 11:**
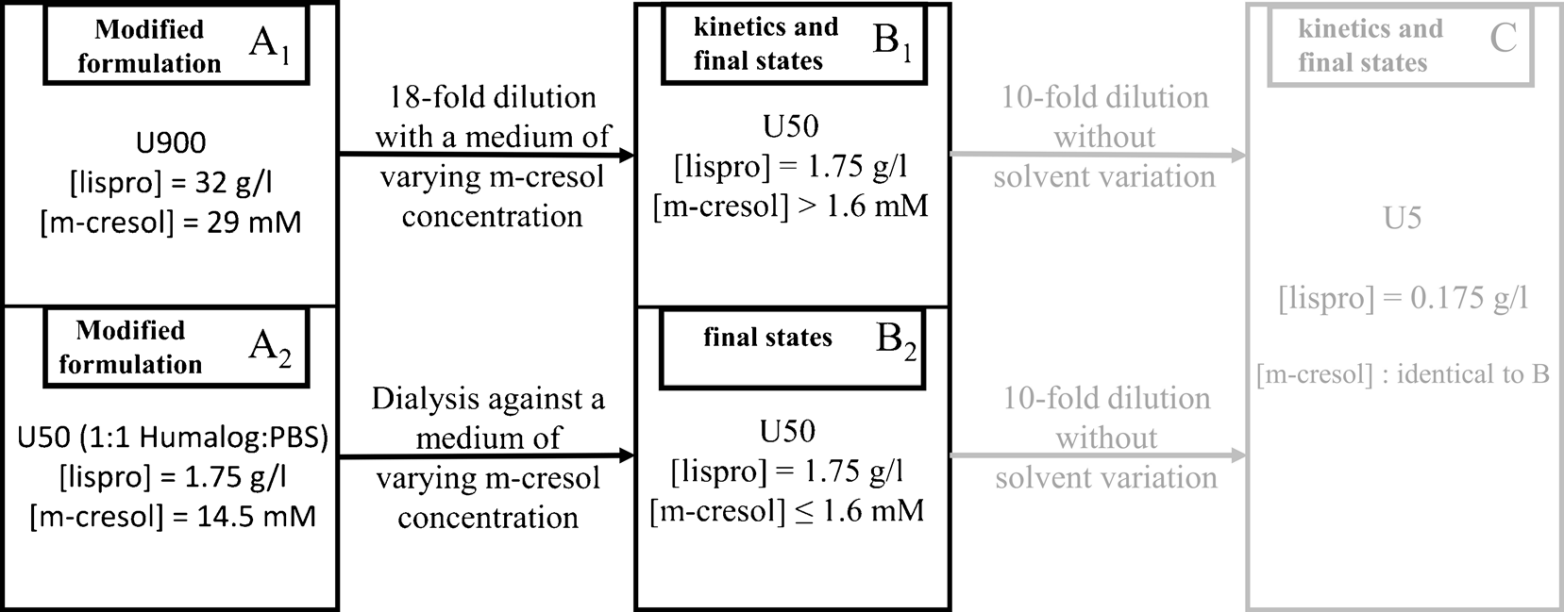
Visualization of experimental scheme II for investigations imitating the processes after subcutaneous injection. U50 concentrations (B1 and B2) with defined m-cresol concentrations were obtained starting from two initial formulations A1 and A2 via dilution or dialysis using media with varying m-cresol concentration, respectively. The concentrations of all other solution components were kept constant and correspond to a twofold dilution of the respective formulation with PBS.

The results presented in the previous section have demonstrated the importance of the concentration of phenolic additives for the dissociation rates of the insulins lispro and aspart. Since the disappearance rate of phenolic compounds in the ST is rather high, we have applied the experimental scheme II (Fig. 11) to study the influence of decreasing m-cresol concentration on the dissociation process of lispro after twofold dilution of Humalog® U100 with PBS. Humalog® was chosen, because lispro approaches an essentially monomeric state in the absence of Zinc and phenolic additives at concentrations as high as 1.75 g/l (see Fig. 1a). In addition, we also tested whether a decrease in m-cresol concentration changes the association state of glulisine under similar conditions.The experiments with Humalog® demand a fast solvent exchange leaving the insulin concentration nearly constant. This is not trivial if one is interested in measuring the kinetics of dissociation. A method to study at least equilibrium states is dialysis against media with varying m-cresol concentration, but otherwise unchanged solvent composition. In order to circumvent the problems involved in measuring kinetic data, we have developed a strategy allowing both kinetic and equilibrium experiments, which is based on scheme II described in materials and methods. The entire experimental strategy is shown in Fig. 11. A_1_ and A_2_ denote the initial conditions with18-fold dilution or dialysis, respectively. B_1_ and B_2_ are the final conditions, which differ only in the concentration range of m-cresol. The attainable concentration range for B_1_ is above 1.6 mM.

**Fig. 12:**
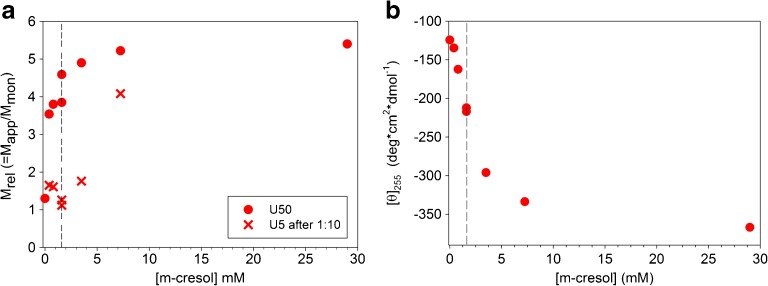
Plots of (**a**) M_rel_ and (**b**) [θ]_255_ of lispro versus m-cresol concentration after processing of samples according to experimental scheme II. M_rel_ was determined at lispro concentrations of U50 (circles) and U5 (crosses). Values at 0 mM and 29 mM m-cresol represent insulin dissolved in PBS and formulation, respectively. The dashed lines separate data obtained by dialysis and dilution.

After each dilution step from A_1_ to B_1_, we have measured the kinetics of dissociation in terms of the light scattering intensity, Stokes radius, and CD signal at 255 nm and the final equilibrium data. The time constants (τ_1_) for approaching the equilibrium states calculated using single-exponential fits are given in Table III. M_rel_ of the final association states at different m-cresol concentrations obtained by either dilution or dialysis are shown in Fig. 12a and Table III. Results from dilution and dialysis are separated by the dashed line in Fig. 12a. The values of M_rel_ at 0 and 29 mM m-cresol are those attributed to PBS and U100 formulation, respectively (cf. Fig. 1a and b). Table III also contains the equilibrium Stokes radii of lispro in the B states. Comparable results at 1.6 mM m-cresol confirmed the equivalence of the two pathways A1 → B1 and A2 → B2 (Fig. 12a, b and Table III).

**Table III:** Summary of Experimental Data Acquired Using Scheme II

		A → B (U50)				B → C (U5)			
	[m-cresol] (mM)]	M_rel, 1_	R_S, 1_ (nm)	τ_1, LS_ (min)	τ_1, CD_ (min)	M_rel, 2_	R_S, 2_ (nm)	τ_2, LS_ (min)	τ_2, CD_ (min)
18-fold dilution of U900	7.3	5.2	2.51	32	25	4.1	2.46	57.0	**
	3.5	4.9	2.48	3.6	2.8	1.8	1.75	22.0	23.3
	1.6	4.6	2.52	(0.5)	(1.0)	1.1	1.43	3.1	5.0
Dialysis of U50	1.6	3.9	2.44			1.3	1.45	3.0	2.2
	0.8	3.8	2.53			1.6	1.44	1.6	(0.9)
	0.4	3.6	2.63			1.7	1.33	*	*

*too fast to measure τ properly

**n.d. due to very small signal changes

Estimated errors are about 8% for M_rel_, 2% for R_S_ and 20% for τ. In the cases these errors are exceeded, values are given in parentheses

Fig. 12b shows the specific ellipticities at 255 nm in the final states B. Again, the values at 0 and 29 mM m-cresol are those corresponding to PBS and U100 formulation, respectively.

It is instructive to compare the CD signals and M_rel_ at low m-cresol concentrations. While the CD signal hints at a preferential population of the monomeric state, M_rel_ remains rather large (close to 4). Furthermore, there are practically no changes in R_S_ (see Table III). Both, the values of M_rel_ and R_S_ point to the existence of an oligomeric state. Thus, it was essential to investigate the kinetic stability of the association states B using 1:10 dilution jumps under constant solvent condition (Fig. 11, B → C).

M_rel_ after additional 1:10 dilution is shown in Fig. 12a. The 10-fold dilution was chosen in order to reach the same final insulin concentration as in 20-fold dilution experiments of U100 using scheme I. Note that the final solvent conditions resulting from scheme I and II setups are different. The measured time constants (τ_2_) and the corresponding equilibrium values of M_rel_ and R_S_ are given in Table III.

For comparison, we have investigated the influence of m-cresol on the association state of glulisine. As the transitions are always fast in the case of glulisine (see Fig. 3), we have measured only M_rel_ and R_S_ in equilibrium states at different m-cresol concentrations. M_rel_ changed from 4.1 to 3.0 upon lowering the m-cresol concentration from 14.5 mM down to 1.9 mM (data not shown). The values are between those, which could be expected as limits from the data shown in Fig. 1a and c for PBS and diluted formulation, respectively.

## Discussion

The focus of this work is to compare the dissociation kinetics of commercially formulated rapid-acting insulins and human insulin. A description of the differences should be helpful to reveal influences and basic mechanisms that are important for an understanding of the entire absorption process of insulins in the patient after injection.

The pharmacokinetic behavior of marketed RAI formulations has been studied demonstrating that insulin glulisine is absorbed slightly faster compared to insulin lispro and insulin aspart (31,32). Surprisingly, the differences in dissociation kinetics observed in our experiments are significantly more pronounced. This underlines that the absorption of an RAI is not solely governed by its dissociation behavior. We additionally demonstrate that the amount of phenolic compounds present in solution strongly influences insulin dissociation times at conditions expected after subcutaneous injection. However, for a complete understanding of the RAI pharmacokinetics, a thorough analysis of all processes involved in the absorption process is essential.

### Comparison of Specific Equilibrium Association States of RAI and HI

The concentration dependences observed under formulation conditions and in PBS yield the association equilibria, which are measures of the stability of oligomeric structures, under the limiting conditions of sufficiently high concentrations of stabilizing ligands and of their virtual absence.

The formation of classical oligomeric structures in PBS (Fig. 1a) is restrained because of the absence of ligands like Zn^2+^ ions (22,33). All RAI form considerably smaller assemblies than HI particularly at higher insulin concentrations. Moreover, the tendency to associate in the absence of Zn^2+^ is different for the various RAI. These observations demonstrate that the particular modifications in the primary structure result in altered subunit interactions. The association state at low concentrations is closest to the monomeric one in the case of insulin lispro. This is in agreement with previous observations (13).

Here, we use the notation ‘assemblies’ for all types of oligomers, for which the structural arrangement of subunits (i.e., the quaternary structure) is not fully verified. We refrain from modeling the experimental data using association equilibria between specific oligomers, as they exist in the presence of Zn^2+^. It has been shown for equilibrium centrifugation (7) and static light scattering data (34) obtained for bovine insulin in the absence of Zn^2+^ that the results can be fitted by simple isodesmic infinite or enhanced isodesmic infinite self-association models. The simple isodesmic infinite association model is based on a stepwise addition of monomers to each existing oligomer with identical association constant. However, these association schemes can be ruled out, if an upper limit of oligomer size is approached above a characteristic insulin concentration.

Such a behavior was indeed observed for lispro, aspart and HI in formulation (Fig. 1b). Lispro, aspart and HI are in the hexameric state throughout the investigated concentration range. The conformation of the hexamers is assumed to be R_6_, since both Zn^2+^ and phenolic additives are present in all formulations. The corresponding near-UV CD spectra (Fig. 4), particularly the strongly negative ellipticities between 251 and 255 nm, support this assumption.

The association equilibrium of glulisine in its formulation lacking Zn^2+^ is very different and resembles its association equilibrium in PBS. The stabilization of larger assemblies due to the presence of m-cresol is clearly visible, but the assemblies of glulisine in its formulation are much less stable than the oligomers of lispro, aspart and HI in their respective formulations containing Zn^2+^.

Notably, the average size of glulisine assemblies approaches that of hexamers at the concentration used for U100 formulations (c = 3.5 mg/ml, Fig. 1b) and remains essentially constant at higher peptide concentrations (Fig. 10). Additionally, the compactness of glulisine assemblies under formulation conditions is practically indistinguishable from that of the other insulins in their formulations (Fig. 7). Thus, although isodesmic infinite association may be valid for HI in the absence of Zn^2+^, insulin glulisine seems to form well-ordered hexameric assemblies in the presence of m-cresol, but in absence of Zn^2+^. As the CD spectra of glulisine assemblies (Fig. 8) clearly differ from those observed for all investigated insulins in the presence of Zn^2+^ and phenolic ligands, the conformation of glulisine hexamers must be different from the R_6_ state. High-resolution NMR studies would be required to decide, whether it is similar to or different from the well-known T_6_ state of HI.

### Fundamental Differences in the Dissociation Rates of the Various Insulins

**Fig. 13:**
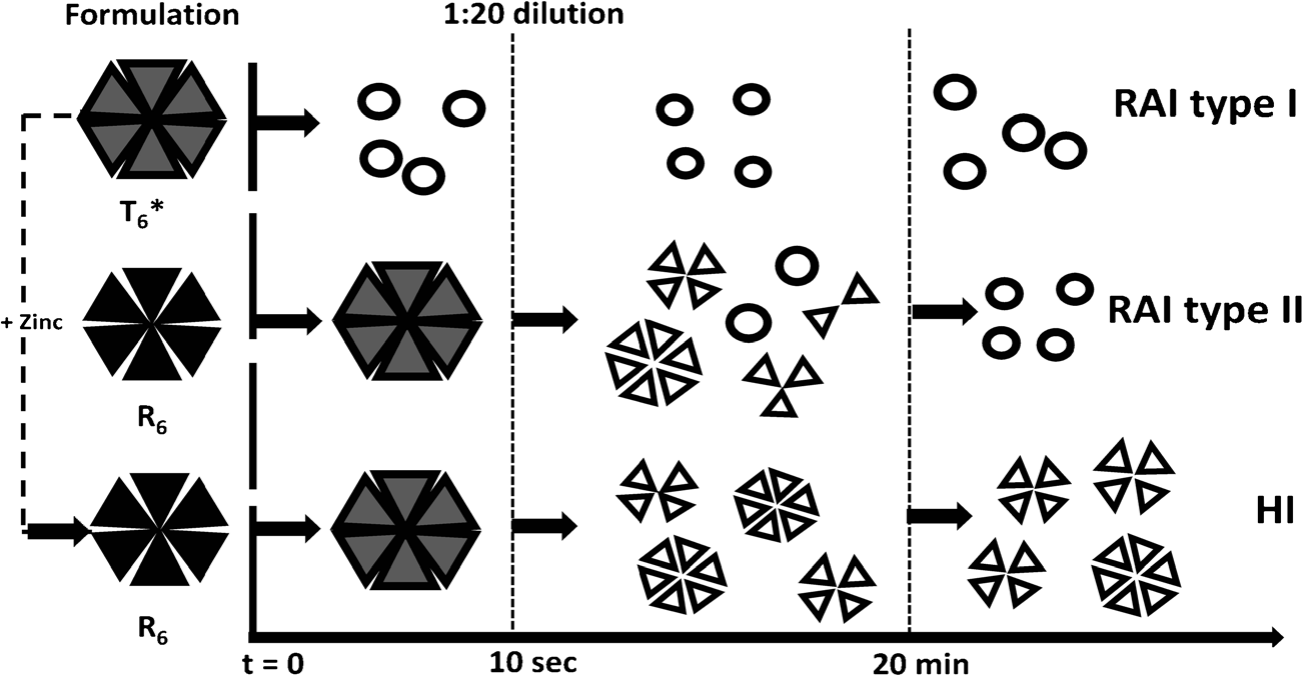
Proposed diagram of states and processes appearing during disassembly of the insulins after 1:20 dilution of formulations with PBS. Triangular symbols depict monomeric subunits assembled into well-coordinated oligomers in the presence of particular ligands (*black*: exclusively R_6_; gray: R_6_, R_3_T_3_ and/or T_6_; not filled: T_6_ or smaller). Open circles represent monomers either isolated or associated to 'oligomeric assemblies'. Upon addition of Zn^2+^, the structure of insulin glulisine in Apidra® (T_6_*) can be converted into R_6_ showing a dissociation behavior like HI. Concerning the classification of the insulins, see main text.

The results of the kinetic light scattering experiments (Fig. 3) not only show clear differences between HI and the RAI, but also between the individual RAI. According to the rates and amplitudes of the kinetic changes, we may classify the insulins investigated here into three categories, as illustrated in Fig. 13: RAI type I, RAI type II and HI. RAI type I stands for the dissociation behavior of insulin glulisine, whereas insulin lispro and insulin aspart are assigned to type II. The initial structures of HI, lispro and aspart are specified as R_6_ hexamers based on their typical near-UV CD spectra (black triangles in Fig. 13).

The initial structure of insulin glulisine symbolized by an assembly of gray triangles in Fig. 13 is predominantly hexameric, and is labeled with T_6_*. The difference in tertiary structure compared to the other insulins is evident from the near-UV CD spectrum. On addition of Zn^2+^, T_6_* hexamers can be converted into R_6_ hexamers comparable to that of HI as indicated by the changes in near-UV CD, dissociation kinetics and association equilibrium at low concentrations (Figs. 8b, 9b and 10). However, these R_6_ oligomers must differ somewhat from their counterparts in the case of HI, lispro and aspart. This is revealed by the formation of larger assemblies (probably di-hexamers etc.) at high glulisine concentrations (Fig. 10). Remarkably, this type of assemblies disappears rapidly within the experimental dead time of the dissociation kinetics (Fig. 9b) reflecting their disassembly prior to the slow hexamer dissociation.

The other arrays of gray triangles in Fig. 13 represents allosteric hexamers, in general. The particular conformation (R_6_, T_3_R_3_ or T_6_) will be a matter of further discussion. The arrays of open triangles in the right interval symbolizes T_6_ hexamers or putative tetramers and dimers being in equilibrium with T_6_ hexamers. Other types of oligomers are shown as assemblies of monomers (open circles).

While the association states of insulin lispro, insulin aspart, and HI remain essentially unchanged within the experimental dead time of 10 s, glulisine immediately attains a completely monomeric state. The rates of changes in the association state observed in the second phase as illustrated in Fig. 13 for lispro, aspart, and HI are similar (Table I). Insulin lispro and aspart approach the monomeric state less completely (M_rel_ = 1.5, Table I) than insulin glulisine. The large final state of HI (M_rel_ = 4.2, Table I) is in accordance to that measured in equilibrium experiments (Fig. 1c) and underlines the high stability of the hexamers.

Equivalent to the observed kinetic changes in the association state, similar rates were observed also for the CD signals of lispro, aspart, and HI within the observable interval (Fig. 5, Table I). In contrast to the corresponding changes in the association state, significant dead time amplitudes were observed, particularly in the case of aspart. The changes of the CD signal of glulisine occurred entirely within the dead time of the experiment.

### Influence of Phenolic Additives and the Mechanisms of Slow Hexamer Dissociation

The influence of formulation excipients on the rates of the transitions is essential for understanding the dissociation mechanisms in detail. An increase in the concentration of excipients did not drive the dissociation constants of glulisine into the observable interval, confirming that the association mechanism of glulisine differs from that of the other RAI. Consequently, systematic studies of the effect of excipients on the rate of dissociation have been done for lispro and aspart only.

We have studied the influences of phenolic additives, excess Zn^2+^ and glycerol that are the major constituents of formulations (Supplemental Table I). Glycerol (data not shown) and excess Zn^2+^ (Table II) had practically no influence on the slow dissociation of both lispro and aspart. However, a considerable influence of m-cresol and equimolar mixtures of phenol and m-cresol was found for both lispro and aspart. The concentration dependence of the measured dissociation time constants is shown in Fig. 6, which can be described by an empirical equation of the form *τ*(*c*) = *τ*_0_ + *a* ⋅ *c* ^*b*^, where c is the total molar concentration in mM and a, b, and τ_0_ are fit parameters. τ_0_ can be considered as dissociation time in the absence of phenolic additives and reflects the dissociation kinetics of T_6_ hexamers. We obtained τ_0_ = 0.27 min and τ_0_ = 0.32 min for lispro and aspart, respectively. The values of τ_0_ are in good agreement with the results of lifetime measurements (21) under comparable conditions. We found no dependence on insulin concentration of the dissociation time at high concentrations of phenolic additives. However, a subtle decrease in τ with decreasing insulin concentration at low concentration of m-cresol was found (see Table II).

In the light of earlier publications, the long dissociation times and the strong influence of phenolic excipients on the dissociation rates are not surprising. Birnbaum *et al*. (20) have measured the rate of bivalent metal ion extraction from Co(II)-R_6_ hexamers of lispro and HI. Hassiepen *et al*. (21) have reported results on kinetic lifetimes of T_6_, T_3_R_3_ and R_6_ hexamers. Interestingly, the lifetimes were about one minute for T_6_, but as large as hours for R_6_ hexamers. Further information about the kinetic stability of the Zn(II)- and Co(II)-insulin hexamer complexes has been obtained using the chromophoric chelator 2,2′,2″-terpyridine as a kinetic probe (10,19). Furthermore, these authors have additionally discussed the relation between kinetic and thermodynamic stability in the presence of phenolic ligands and the high kinetic barrier for the transition to T_6_.

Altogether, these results have been obtained using elegant but indirect approaches to measure oligomer dissociation. Taken together, those results and our direct measurements of oligomer size indicate that the association state of type II RAI existing after the dead time (Fig. 13, gray symbols) is still the R_6_ state. The measured dissociation is then a slow transition from the R_6_ to the T_6_ state. After this rate-limiting step, the less stable T_6_ states dissociate rapidly into monomers. A fast dissociation of lispro after removal of phenol was also discussed by Ciszak *et al*. (12) to explain why the formulated analog exhibits rapid time action after injection despite its hexameric conformation in formulation. HI, however, is still in an association equilibrium shifted towards T_6_ hexamers at a comparably reduced concentration of phenolic ligands.

The proposed dissociation kinetics characterized by a rate-limiting transition from R_6_ to T_6_ hexamers, followed by a faster transition towards monomers, is reflected in the slow changes of the CD signals. However, the interpretation of the observed dead time amplitudes in the CD signal at 255 nm of lispro and aspart (less pronounced in the case of HI) is not straightforward. The specific ellipticities approached within the dead time in this wavelength region are comparable with those typical of T_3_R_3_ hexamers. This could be interpreted as a transient population of T_3_R_3_ states on the transitions between R_6_ and T_6_ as it has been discussed frequently. Since conformational transitions from T_3_R_3_ to T_6_ states are slow, the additional population of a T_3_R_3_ state is not in conflict with the observed slow changes in CD and light scattering signals. However, there is a discrepancy concerning the fast rate of population of the T_3_R_3_ state right after dilution of formulation, which must be very high according to the dead time CD amplitudes. This is extremely unlikely since high kinetic barriers between R_3_ and T_3_ are expected in any case because of the conformational switch in the N-terminus of the B-chains for R_6_ → T_3_R_3_ and T_3_R_3_ → T_6_ as well (19).

Changes of the CD signals in the wavelength range between 250 and 255 nm are expected to result from specific variations in the environment of PheB^1^ (8). An alternative explanation is possible concerning rather unspecific effects caused by the drop (mostly 20-fold) in the concentration of phenolic compounds during the applied dilution jumps. Indeed, the concentrations of phenolic substances used in formulation and in our experiments for measuring the effects on dissociation rates are so high that mass action effects of binding can be expected (19). This is also supported by our observation that the dissociation rates depend on the total concentration of phenolic additives and practically not on the molar ratio [phenol]/ [insulin] at high concentrations of phenolic additives. The changes in CD could result from changes in the packing density of the tertiary and quaternary structures upon binding of phenolic additives. Such a mechanism was already discussed by Rahuel-Clermont *et al*. (19). A precondition for further discussion of the dead time CD changes is to assemble complete CD spectra from single kinetic records at several wavelengths.

### Impact of Phenolic Additives on the Dissociation Behaviour under Subcutaneous Injection-like Conditions

For a rapid absorption of insulin after subcutaneous injection, a fast dissociation of insulin hexamers is necessary (35). The disappearance rate of phenolic additives is supposed to be one rate-limiting factor in this process (12,23). Yet, it is not known, how the acceleration of dissociation proceeds in detail and how different insulin analogs respond to a decrease in the concentration of phenolic additives. In the following, we will discuss the results of twofold dilution of lispro (Humalog®) as a prototype for the behavior of the RAI type II insulins. The results concerning the RAI type I insulin glulisine have confirmed the expected small effect of decreasing concentrations of m-cresol on the association state and do not demand detailed discussion.

Kinetic changes upon lowering the m-cresol concentration were monitored by measuring the time courses of the light scattering intensity and the CD signal at 255 nm (see Table III). Detectable changes of the association state could be observed only when the concentration was lowered at least down to 7.3 mM m-cresol, where we obtained time constants of τ_1,LS_ = 25 min and τ_1,CD_ = 32 min. Further dilution down to 3.5 mM led to a nearly 10-fold decrease in the dissociation time constants revealing a steep concentration dependence in this range. Thus, right after injection at m-cresol concentrations of 14.5 mM the dissociation times must be in the order of hours.

At m-cresol concentrations below ~2 mM, lispro is able to dissociate quickly within a few minutes. Accordingly, the dissociation rate should be no longer a bottleneck for the absorption process. Under these conditions, both type I and type II RAI dissociate rather fast. This explains why the extremely faster dissociation kinetics of glulisine is only marginally translated into the pharmacokinetic profile.

Interestingly, the concentration of about 2 mM is close to the midpoint of the transition of the CD signal at 255 nm, which indicates the transition from the hexameric R_6_ state to the monomeric state (Fig. 12b). The transition to the monomeric state appears to be almost complete at the lowest m-cresol concentration of 0.4 mM used in our studies (Fig. 12b). This was supported by the CD spectrum (data not shown), which was essentially identical to that typical of the monomeric state of lispro (Fig. 4a).

In contrast to these observations, M_rel_ does not considerably decrease with m-cresol concentration. Particularly at 0.4 mM m-cresol, lispro is still in an association state far from the monomeric one. This contradiction hints to the formation of an alternative type of assemblies, which involves monomeric subunits with the structure of isolated monomers. The formation of higher order assemblies upon depletion of phenolic additives has been described also by Teska *et al*. (36). The association states may resemble rather colloidal assemblies than discrete (classical) insulin oligomers.

However, irrespective of the structure of the intermediate association states at low m-cresol concentrations, their ability to dissociate is more important. For this purpose, we have done additional 1:10 dilution experiments with all B state samples (Fig. 11). Below 1.6 mM m-cresol, lispro is preferentially in the monomeric state (Fig. 12a). The equilibrium state is approached with τ_2_ < 5 min (Table III). This means that the formation of these transient association states does not considerably slow down the dissociation process.

The situation is different at high m-cresol concentrations. At 7.3 mM, M_rel_ changes only from 5.2 to 4.1 with τ_2_ = 57 min. This means that the majority of lispro molecules remains still in the original R_6_ state and only a small fraction dissociates slowly.

In summary, with our experimental strategy we were able to determine dissociation rates and the attained equilibrium association states at discrete m-cresol concentrations. However, without information about *in vivo* distribution rates of phenolic additives, we cannot predict actual dissociation times of insulins in the IF.

It is tempting to speculate, which additional factors other than the prolongation of dissociation rates by phenolic additives are time limiting for absorption. According to our results (Fig. 1c), the decrease in insulin concentration itself must be equally important by shifting the equilibrium towards dimers and monomers. It has to be taken into account that the decrease in the instantaneous insulin concentration due to diffusion within the subcutaneous tissue and by penetration into capillaries is a particular driving force for dissociation thus accelerating the dissociation in a feedback-loop manner. In this context, faster acting insulin formulations must improve the transition efficiency of insulins from ST into the bloodstream. Accordingly, additional excipients could be tested for this purpose as recently shown by Heise *et al*. (15,16) in the case of aspart. It would be interesting to investigate if the used excipient nicotinamide (probably responsible for acceleration) also alters the absorption properties of other RAI formulations.

## Conclusions

Our investigation is the first direct comparison of the inherent dissociation properties of marketed fast-acting insulins.

Despite the essentially accelerated dissociation of all three RAI compared to HI, there are distinctive differences between glulisine (type I RAI) and lispro and aspart (type II RAI). From a biophysical point of view, these are mainly based on the different kinetic stabilities of the insulins at their specific formulation conditions.

The superior dissociation behavior of glulisine is based on its specific oligomeric state in formulation lacking Zn^2+^. The low kinetic stability of this essentially hexameric state compared to that of the R_6_ state existing in formulations containing both Zn^2+^ and phenolic additives ensures very fast dissociation. The time constants are shorter than our experimental dead time of 10 s under all conditions obtained after dilution of formulation.

The dissociation behavior of type II RAI is affected by the high stability of the hexameric R_6_ state in the formulations containing Zn^2+^ and phenolic additives. However, an acceleration of dissociation is attainable by fast depletion of the concentration of phenolic additives right after dilution. Similar conditions exist after subcutaneous injection due to a fast diffusion of phenolic additives. Our investigations with Humalog® revealed that a tenfold reduction in m-cresol concentration decreases the dissociation time from about one hour to one minute. Consequently, also the dissociation of type II RAI becomes fast enough not to be the limiting factor of the absorption process.

A further acceleration of insulin absorption is obviously regulated by other factors as diffusion in the ST or the transition from ST into blood capillaries. The decrease in the actual insulin concentration directly influences the dissociation equilibrium and is thus an additional driving force for dissociation.

In this context, our results will be helpful to optimize future formulation conditions for rapid-acting insulins.

## Electronic supplementary material

ESM 1 (DOCX 16 kb)

## Abbreviations

_θ_: Ellipticity
_[θ]_: Mean residue ellipticity
_τ_: Dissociation time constant
ACF: Autocorrelation function
c: Insulin concentration in mg/ml
CD: Circular dichroism
D: Translational diffusion coefficient
DLS: Dynamic light scattering
dn/dc: Refractive index increment
EDTA: Ethylenediaminetetraacetic acid
HI: Human insulin
I: Scattering intensity
IF: Interstitial fluid
K_opt_: Optical constant
M_app_: Apparent molecular mass
M_mon_: Molecular mass of insulin monomers
M_rel_: Relative apparent molecular mass
RAI: Rapid-acting insulin
R_S_: Stokes radius
SLS: Static light scattering
(S/N): Signal-to-noise
ST: Subcutaneous tissue
T_acc_: Accumulation time
Ux: x Units/ ml insulin
