# Supplementary material for: Rapid-Acting and Human Insulins: Hexamer Dissociation Kinetics upon Dilution of the Pharmaceutical Formulation
Source: Pharm Res. 2017 Jul 31;34(11):2270–86. doi: 10.1007/s11095-017-2233-0 (PMC5643355; doi:10.1007/s11095-017-2233-0)
Supplement: Supplementary file 1 — (DOCX 16 kb) [file 11095_2017_2233_MOESM1_ESM.docx]

**Supplemental Table I: Composition of formulations**

The compositions of Humalog^®^ and NovoRapid^®^ were taken from <http://www.rxlist.com>, the ones of Apidra^®^ and Insuman Rapid^®^ were provided by Sanofi-Aventis Deutschland GmbH

| formulation | insulin | components in formulation (U100) | | | | | |
| --- | --- | --- | --- | --- | --- | --- | --- |
|  |  | buffer | Zn^2+^ | NaCl | m-cresol | phenol | Glycerol* |
| Humalog^®^ | 0.6 mM lispro  (= 3.5 g/l) | 7 mM phosphate, pH 7.4 | 0.3 mM | -- | 29 mM | -- | 18.8 g/l |
| NovoRapid^®^ | 0.6 mM aspart  (= 3.5 g/l) | 7 mM phosphate,  pH 7.4 | 0.3 mM | 10 mM | 16 mM | 16 mM | 18.8 g/l |
| Apidra^®^ | 0.6 mM glulisine  (= 3.5 g/l) | 50 mM Tris,  pH 7.3 + Tween 20 | -- | 86 mM | 29 mM | -- | -- |
| Insuman Rapid^®^ | 0.6 mM HI  (= 3.6 g/l) | 8 mM phosphate,  pH 7.3 | --** | -- | 25 mM | -- | 18.8 g/l |

*: 85% glycerol, ** insulin contains Zn^2+^, but no further added Zn^2+^

**Supplemental Table II: Key components of physiologic dilution media**

|  | PBS* | Interstitial Fluid^1^ |
| --- | --- | --- |
| Total Protein | - | 20.6 g/l |
| Na^+^ | 140 mmol/L | 135 mmol/L |
| K^+^ | 3 mmol/L | 4 mmol/L |
| Ca^2+^ | - | 1.2-1.6 mmol/L |
| Mg^2+^ | - | - - - 1. mmol/L |

* PBS (10 mM phosphate buffer, pH 7.4 tablets from Merck (Calbiochem)

Reference

1: Fogh-Andersen N, Altura BM, Altura BT, Siggaard-Andersen O. Composition of

interstitial fluid. Clin Chem. 1995 Oct;41(10):1522-5.
